# *In silico, in vitro*, and *in vivo* Approaches to Identify Molecular Players in Fragile X Tremor and Ataxia Syndrome

**DOI:** 10.3389/fmolb.2020.00031

**Published:** 2020-03-11

**Authors:** Saif N. Haify, Teresa Botta-Orfila, Renate K. Hukema, Gian Gaetano Tartaglia

**Affiliations:** ^1^Department of Clinical Genetics, Erasmus MC, Rotterdam, Netherlands; ^2^Biological Fluids Bank of the Institut d’Investigacions Biomèdiques August Pi i Sunyer (IDIBAPS), Barcelona, Spain; ^3^Centre for Genomic Regulation (CRG), The Barcelona Institute for Science and Technology, Barcelona, Spain; ^4^Institució Catalana de Recerca i Estudis Avançats (ICREA), Barcelona, Spain; ^5^Department of Biology ‘Charles Darwin’, Sapienza University of Rome, Rome, Italy; ^6^Department of Neuroscience and Brain Technologies, Istituto Italiano di Tecnologia, Genoa, Italy; ^7^Universitat Pompeu Fabra (UPF), Barcelona, Spain

**Keywords:** RNA, Fragile X associated tremor ataxia syndrome (FXTAS), mouse model, computational modeling, protein network

## Abstract

Fragile X-associated tremor/ataxia syndrome (FXTAS) is a late-onset neurodegenerative monogenetic disorder affecting carriers of premutation (PM) forms of the *FMR1* gene, resulting in a progressive development of tremors, ataxia, and neuropsychological problems. This highly disabling disease is quite common in the general population with an estimation of about 20 million PM carriers worldwide. The chances of developing FXTAS increase dramatically with age, with about 45% of male carriers over the age of 50 being affected. Both the gene and pathogenic trigger, a mutant expansion of CGG RNA, causing FXTAS are known. This makes it an interesting disease to develop targeted therapeutic interventions for. Yet, no such interventions are available at this moment. Here we discuss *in silico*, *in vitro*, and *in vivo* approaches and how they have been used to identify the molecular determinants of FXTAS pathology. These approaches have yielded substantial information about FXTAS pathology and, consequently, many markers have emerged to play a key role in understanding the disease mechanism. Integration of the different approaches is expected to provide crucial information about the value of these markers as either therapeutic target or biomarker, essential to monitor therapeutic interventions in the future.

## Introduction

Fragile X-associated Tremor and Ataxia syndrome (FXTAS) is a late onset neurodegenerative disease that affects Fragile X premutation (PM) carriers. The Fragile X gene (*FMR1*) responsible for the disease finds its origin on the X chromosome and codes for the Fragile X Mental Retardation Protein called FMRP. This protein is essential in the neuronal development and synaptic plasticity of the brain. The 5′ untranslated region (UTR) of the *FMR1* gene carries a variable number of CGG repeats, which is between 4 and 55 in healthy individuals. Due to the instability of the repeat the CGG sequence can expand to 55–200 repeats over generations (Leehey, 2009; Berman et al., 2014; Foote et al., 2016; Glineburg et al., 2018). These individuals are referred to as PM carriers.

**FIGURE 1:**
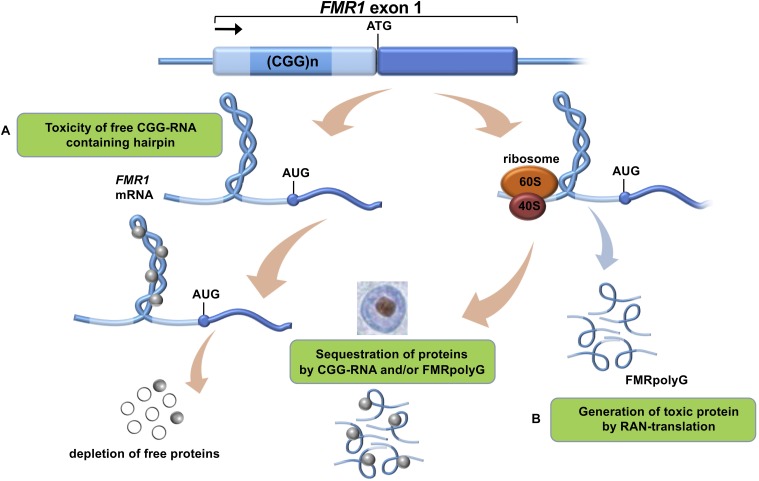
Proposed mechanisms of CGG-repeat toxicity in PM carriers. **(A)** Protein sequestration model: RNA binding proteins are sequestered through their interactions with the expanded CGG-repeat *FMR1* mRNA. These proteins can in turn recruit other proteins. The net result of the sequestration of these proteins is that they are unavailable to carry out their normal functions and critical cellular processes are thereby altered or blocked. **(B)** Toxic polypeptide model: the ribosome translation initiation complex stalls near the CGG repeat hairpin formed on the *FMR1* mRNA. This promotes the repeat-associated non-AUG (RAN) translation of *FMR1* mRNA using a near-AUG start site. This results in a frame shift and the production of the polyglycine-containing polypeptide (FMRpolyG) that somehow interfere with normal cell function or may be directly toxic.

FXTAS is characterized by several clinical features such as intention tremors and cerebellar gait ataxia, which are proposed to be major clinical diagnostic criteria for FXTAS disease pathology. Other, more minor clinical criteria are Parkinsonism, working memory deficit and executive function deficit (Berry-Kravis et al., 2007; Leehey, 2009; Berman et al., 2014). Furthermore the neurodegeneration is characterized by brain atrophy, neuropsychiatric features and cognitive impairments or dementia (Foote et al., 2016). It is estimated that in the general population 1:110-250 females and 1:260-800 males are PM carriers with an interesting side note that not all carriers develop FXTAS due to incomplete penetrance (Tassone et al., 2012; Hunter et al., 2014; Foote et al., 2016; Glineburg et al., 2018). FXTAS penetrance is about 40% in male carriers and only 11–18% in female carriers (Berry-Kravis et al., 2007; Foote et al., 2016; Glineburg et al., 2018). A major hallmark of the disease is the presence of ubiquitin-positive intranuclear inclusions throughout the brains of FXTAS patients (Figure 1).

To date there is no cure available for PM carriers or FXTAS patients which is why development and validation of relevant models is necessary (Leehey, 2009). This will allow us to better understand this complex neurological disorder and also to be used for drug development in the future. At present, the onset and development of FXTAS is explained by two main mechanisms (Botta-Orfila et al., 2016): (i) RNA-mediated sequestration, and subsequent inactivation, of proteins attracted by the CGG trinucleotide repeats in the 5′ UTR region of *FMR1* RNA and (ii) toxic aggregation of Repeat-Associated Non-AUG (RAN) polyglycine peptides translated from the *FMR1* 5′ UTR (FMRpolyG; Figure 1). Previous work indicates that *FMR1* RNA forms aggregates containing specific proteins such as HNRNP A2/B1, MBNL1, LMNA, and INA (Iwahashi et al., 2006). Also the FMRpolyG peptide (Sellier et al., 2017) was found in the aggregates, together with CUGBP1, KHDRBS1, and DGCR8 being involved in splicing regulation, mRNA transport and regulation of microRNAs (Sellier et al., 2010).

Here we discuss *in silico*, *in vitro*, and *in vivo* approaches and how they have been used to identify the molecular determinants of FXTAS pathology. Our aim is to provide a comprehensive review for future research in the area. We believe that synergetic approaches from different research areas are necessary for FXTAS, because its pathological substrate is still under debate and there is still insufficient knowledge of targets for the development of a therapeutic intervention.

## *In Silico* Approaches to Predict Molecular Interactions Occurring in FXTAS

Difficulties in the biochemical purification of protein–RNA assemblies, which are extremely labile, make it extremely hard to extract the aggregates and identify the molecules that are crucial in disease spreading (Tartaglia, 2016). The use of computational methods aids in characterizing RNA-binding proteins (RBPs) with CGG repeats. We refer the reader to advanced reviews for more details related to some sophisticated algorithms (Cirillo et al., 2014).

## Predictions of Protein–RNA Interactions Occurring in FXTAS

The ability of the first *FMR1* exon (containing 79 CGG repeats in the PM range) to interact with specific RBPs. was assessed through the *cat*RAPID algorithm (Agostini et al., 2013). *cat*RAPID estimates the binding potential through van der Waals, hydrogen bonding and secondary structure propensities of both protein and RNA sequences allowing identification of binding partners (Bellucci et al., 2011). The library employed in the study was composed of 3340 DNA-binding, RNA-binding, and structurally disordered proteins (Livi et al., 2016).

**FIGURE 2:**
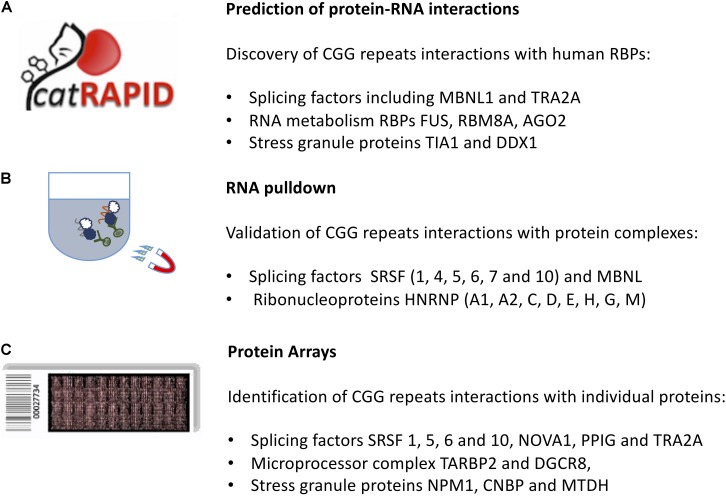
*In silico* and *in vitro* methods to identify protein interactors of expanded CGG repeats in the *FMR1* mRNA. **(A)** The *cat*RAPID approach can be used to predict the protein partners of expanded CGG repeats exploiting calculations of secondary structure and a phenomenological potential for van der Waals and hydrogen-bonding interactions; **(B)** RNA pull-down followed by mass-spectrometry reveals the most abundant protein interactors of *FMR1* 5′ UTR. **(C)** The protein microarray approach allows to probe labeled *FMR1* 5′ UTR against the entire human proteome, revealing targets that are poorly abundant in the cell.

*cat*RAPID (Figure 2) identified known CGG-binding proteins such as ROA (1, 2, and 3), SRSF (1, 4, 5, 6, 7, and 10), HNRNP (C, D, M) as well as MBNL1 and KHDRBS3 (Cid-Samper et al., 2018) (see section “*In vitro* Approaches to Predict Molecular Interactions Occurring in FXTAS”). In addition, strong binding propensities were found for an additional set of 92 proteins that are known to aggregate in stress granules (Jain et al., 2016) and a group of 37 RBPs that have strong potential to aggregate, as predicted by the *cat*GRANULE algorithm (Bolognesi et al., 2016). Among the RBPs identified, there are 25 splicing factors, including LSM3, SFPQ and TRA2A, 24 RBPs involved in RNA metabolism, including FUS, RBM8A, AGO2 and stress granules RBPs such as TIA1, MBNL1, and DDX1 (Cid-Samper et al., 2018) as well as CIRBP and PTBP2 (Cirillo et al., 2013).

## *In Vitro* Approaches to Predict Molecular Interactions Occurring in FXTAS

Protein interaction with CGG repeats can be determined by several experimental *in vitro* approaches. The success in discovering new findings is influenced by the technical capacity to preserve the natural characteristics of the protein–RNA partners, such as the secondary and tertiary structure, electrostatic and hydrophobic interactions, hydrogen bonding, rate of transcription of RNA and translation of protein, etc. We refer the reader to advanced reviews for details related to the experimental methods (Dasti et al., 2019).

CGG repeats form hairpins *in vitro* (Krzyzosiak et al., 2012). UV-monitored structure melting indicates that they are more stable than hairpins formed by CAG, CUG, or CCG repeats. Although the *in vivo* structure might differ, due to interactions with proteins and other molecules, crystallography supports the model that CGG repeats have intermolecular duplexes (Kiliszek et al., 2011).

## RNA Pull-Down to Detect CGG Repeats Interactions

The RNA pull-down consists on a selective extraction of a protein–RNA complex from a whole sample lysate, by using high-affinity tags, e.g., biotin on the RNA molecule. After tagging the known interaction partner, RNA, the RBPs complexed with it are purified by using agarose or magnetic beads. To identify which proteins are associated with expanded CGG repeats, Sellier and colleagues adopted an *in vitro* approach. Proteins extracted from mouse brain and COS7 cell nuclei were captured on streptavidin resin coupled to biotinylated *in vitro*-transcribed RNA composed of 60CGG repeats, eluted, separated on SDS–PAGE gels and identified by MALDI-TOF analysis. More than 20 proteins were identified, including a heat-shock protein and several RBPs, such as SRSF (1, 4, 5, 6, 7, and 10), MBNL1 and HNRNP-G (Sellier et al., 2010). The list of interactors (Figure 2) included SPNR, HNRNP-A1, HNRNP-A2/B, HNRNP-C, HNRNP-D, HNRNP-E, and HNRNP-H (Sellier et al., 2010).

## Protein Microarrays to Detect *Fmr1* Interactions

Protein microarray technology was used to detect RBP interactions with the first *FMR1* exon (Cid-Samper et al., 2018). In this approach, individual human proteins, expressed in a eukaryotic system and subsequently purified, are isolated in separated nitrocellulose chambers and the RNA labeled with Cy5 is used for probing (Marchese et al., 2017). Both expanded (79CGG, permutated range) and normal (21CGG) repeats were probed on independent replicas and the 3′ UTR of a similar length transcript, SNCA, was used as a control for the specificity of RBP interactions (Marchese et al., 2017).

Using fluorescence intensities to measure binding affinities (Cirillo et al., 2016), previously identified partners SRSF 1, 5, and 6 ranked in the top 1% of all interactions, followed by KHDRBS3 (2%) and MBNL1 (5%). The overall list included 85 RBPs showing an enrichment in Gene Ontology terms related to splicing activity, as reported by *clever*GO (Klus et al., 2015) and includes several SRSF proteins, PCBP 1 and 2, HNRNP-A0 and F, NOVA1, PPIG, and TRA2A (Figure 2).

## RNA Electrophoretic Mobility Shift Assay (RNA-Emsa) to Validate Individual Protein Interactions With CGG Repeats

In the RNA-EMSA assay protein–RNA interactions are detected as migration differences in gel electrophoresis: the RNA probe is labeled by radioactivity of by fluorescent or chemiluminiscent dyes, and incubated with the whole protein extract from cell lysate, in different concentrations of the first one. In case the RNA is selectively bound to proteins, the electrophoretic band will differ from a negative control, as a migration shift. In this assay, an experimental secondary approach is performed, by assessing competitive binding: an excess of unlabeled RNA is incubated with the binding reaction and in case the shifted signal decreases, there is evidence for specificity of the binding.

Using RNA-EMSA, the binding of HNRNP-A2 to 105CGG repeats was studied in the presence of the brain cytoplasmic (BC1) RNA involved in neuronal translational (Muslimov et al., 2011). Differently from 105CCC repeats, the 105CGG repeats competed with binding of BC1 RNA to HNRNP-A2, which indicates impairment of neuronal function. Using 105CGG repeats at levels comparable to *FMR1* abundance in PM disease cells, the authors showed that distal dendritic delivery of BC1 RNA is significantly reduced while 105CCC repeats had no effect on dendritic BC1 targeting (Muslimov et al., 2011).

## RNase H Protection Approach to Characterize the *FMR1* R-Loop

The RNase H protection approach is used to detect DNA and RNA fragments in cell lysates. RNase H cleaves the target RNA molecule at a specific site hybridized with a DNA probe. In these specific sites, if a protein is bound, the hybridization is blocked, therefore no cleavage by RNase H will occur.

It has been observed that transcription through the GC-rich *FMR1* 5′ UTR region favors formation of a three-stranded nucleic acid structure, composed of a DNA:RNA called R-loop formation, with the nascent RNA assembling with the template DNA strand (Loomis et al., 2014). Using DNA:RNA immunoprecipitation of genomic DNA from cultured human dermal fibroblasts with both normal and PM alleles, the authors reported for *FMR1* R-loop formation As expected for R-loop formation. Treatment with purified recombinant human RNases H1 and H2 eliminated DNA–RNA interaction (Loomis et al., 2014).

## Fluorescent *In Situ* Hybridization Co-Localization (Fish) to Localize CGG Repeats in the Cell

Fluorescent *in situ* hybridization (FISH) co-localization techniques require knowing in advance which RNA and protein interaction will be studied. This technique is commonly used in FXTAS research as it allows verifying where CGG repeats localize with other molecules. By means of FISH coupled to immunofluorescence it has been shown that CGG expansions and TRA2A significantly co-localize in COS7 cells (Cid-Samper et al., 2018). Similarly, co-localization of MBNL1, KHDRBS1 and HNRNP-G within CGG aggregates was observed in COS7 cells (Sellier et al., 2010). By contrast, other *in vitro* identified candidates such as SPNR, HNRNP-A1, HNRNP-A2/B, HNRNP-C, HNRNP-D, HNRNP-E, and HNRNP-H have been shown by FISH to have poor co-localization with CGG repeats (Sellier et al., 2010). Several proteins, including a number of heat-shock proteins, HNRNP-A2/B1, CUGBP1, lamin-A/C and MBP were found to localize with ubiquitin-positive inclusions in CGG-expressing *Drosophila*, KI mouse model and FXTAS patients. Some of the co-localizations are model dependent. Indeed, it should be noted that PURα co-localizes with cytoplasmic CGG repeats in flies (Jin et al., 2007) but not in mammalian cells, where it is found strictly nuclear (Sellier et al., 2010). So, given the propensity of PUR-α to interact with CGG repeats (Cirillo et al., 2013), it is possible that its subcellular localization prevents physical interaction with RNA.

## *In Vivo* Approaches to Predict Molecular Interactions Occurring in FXTAS

The development of animal models has provided the field with important clinical but also molecular information regarding the pathology associated with CGG repeat expansions on *FMR1* Fragile X syndrome (FXS), PM carriers and FXTAS. PM carriers of the expanded CGG repeat have a 2 to 8-fold elevation of *FMR1* mRNA levels when compared to healthy CGG repeat individuals (Hagerman and Hagerman, 2002). Increased levels of *FMR1* mRNA with a CGG expansion are proposed to be toxic due to sequestration of specific RNA binding proteins that are crucial for normal cell function, partly resulting in FXTAS pathology. This theory is supported by inclusions isolated from FXTAS post-mortem brain tissue containing over 30 critical proteins, such as Lamin A/C, KHDRBS1, Drosha, and HNRNP-A2 (Glineburg et al., 2018). The most prominent neuropathological hallmark of FXTAS is the presence of eosinophilic, ubiquitin-positive intranuclear inclusions in neurons and astroglia in the entire brain upon post-mortem histological analysis (Greco et al., 2002, 2006, 2007, 2008; Gokden et al., 2009). Additional neuropathological features found in FXTAS are reduced number of Purkinje cells, Bergmann gliosis, axonal swelling in the granular cell layer of the cerebellum, and prominent cortical and subcortical white matter pathology (Greco et al., 2002, 2006). A second mechanism more recently found suggests an additional model for toxicity in FXTAS called repeat-associated non-AUG (RAN) translation. This mechanism proposes that the expanded CGG repeat is translated in absence of a canonical start codon resulting in pathogenicity through translation of a toxic polyglycine (FMRpolyG) peptide (Berman et al., 2014; Glineburg et al., 2018). Over the past 10 to 15 years many well-characterized mouse models have shown that aforementioned theories could indeed contribute to disease pathology. These mouse models also allowed us to characterize FXTAS disease pathogenesis and progression together with the underlying neurobiological changes and to develop and test highly potential targeted therapeutic interventions. This part of the review will describe the current available mouse models for FXTAS with their specific aspects, advantages and limitations, and what insights they have provided over the past years into disease mechanism.

## The Dutch Mouse

**FIGURE 3:**
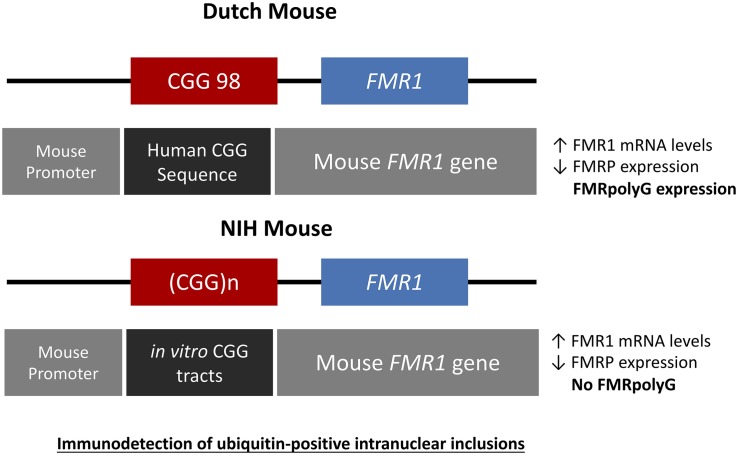
Schematic drawings representing the genetic constructs designed for the Dutch and NIH CGG KI PM mouse models. The Dutch mouse model has an intact mouse promoter followed by a human genetic sequence flanking the inserted CGG repeat expansion upstream of the mouse *Fmr1* gene. The NIH CGG KI mouse has an *in vitro* generated CGG repeat tract inserted to replace the mouse CGG8 also keeping the mouse *Fmr1* gene and promoter intact. In both cases there is immunodetection of ubiquitine-positive inclusions.

The first mouse model generated to exhibit much of the pathology seen in affected PM carriers and in FXTAS pathology at the genetic level but as well as histopathological and molecular level was the Dutch mouse (CGG_dut_ KI) model. This mouse model was developed in Rotterdam at the Erasmus MC in the Netherlands. This model was generated by replacing the endogenous murine *Fmr1* 8CGG repeat with a human 98CGG repeat containing the human *FMR1* flanking regions. The *Fmr1* mouse promoter was left unchanged. This was done by homologous recombination in embryonic stem (ES) cells (Bontekoe et al., 2001; Foote et al., 2016). Upon paternal and maternal transmission the CGG_dut_ KI mouse showed mild instability of the CGG repeat with both short expansions and contractions present (Bontekoe et al., 2001; Willemsen et al., 2003; Brouwer et al., 2007). The CGG_dut_ KI mice (Figure 3) have been bred into a C57BL/6J and FVB background over several generations to establish lines with expanded alleles greater than 450CGGs (Bontekoe et al., 2001; Berman and Willemsen, 2009). Although repeat lengths of more than 450CGGs were found, no increased methylation of the *Fmr1* gene has been reported. When examining these mice at the histopathological level clear ubiquitin-positive intranuclear inclusions could be shown similar to what is seen in FXTAS post-mortem patient brain. As mentioned before FXTAS patients are characterized with elevated *FMR1* mRNA and slightly decreased FMRP protein. Although FXTAS is considered to be a late-onset neurodegenerative disorder, some phenotypes of the disease in patients and in the CGG_dut_ KI mouse could suggest that FXTAS might also have features of a neurodevelopmental disorder. Developmental abnormalities during the PM stage such as altered learning and memory may contribute to the late manifestation of FXTAS. For example PM carriers have a smaller hippocampus that correlates with impaired performance in standardized tests of memory (Hagerman, 2013). Hippocampal neurons with an abnormal dendritic morphology have been observed in FXTAS neurons at a time of development when nuclear inclusions are not detectable yet. When observing hippocampal neurons from the CGG_dut_ KI mouse researchers found cortical migration to be affected in these mice and that these neurons upon culturing display shorter dendrites and have a reduced dendritic complexity (Hunsaker et al., 2009, 2012; Chen et al., 2010; Cunningham et al., 2011; Berman et al., 2014). Recently, the impact of elevated *Fmr1* mRNA levels on the morphology of dendrites and axons was studied more in depth (Drozd et al., 2019). Indeed, these morphological phenotypes are associated with increased levels of *Fmr1* mRNA because upon treatment with shRNAs specifically targeting the *Fmr1* mRNA these phenotypes are rescued. In addition, proteomic analysis in the CGG_dut_ KI mouse showed that upon rescue of *FMR1* mRNA levels a large number of important RBPs such as Tia1, HNRNPll, and ROAA could be normalized. Other rescued proteins are Rab-GTPases, which are critical for synaptic function in neurons in brain developmental disorders. Also Aldh4a1/P5CDH and Samm50, which are involved in mitochondrial dysfunction, were found to be deregulated in these mice confirming previous hypotheses indicating the important role of mitochondria in FXTAS pathology. All these proteins provide possible future pharmacological targetable molecules for early therapeutic intervention for FXTAS. More research focusing on other aspects of neurodevelopment in FXTAS such as the role of FMRP in the PM stage is necessary before one can categorize FXTAS to also be a neurodevelopmental disorder. All together, the CGG_dut_ KI mouse model nicely recapitulates the histopathology and molecular changes observed in patients (Berman and Willemsen, 2009; Foote et al., 2016).

## The NIH Mouse

The National Institutes of Health developed a second KI mouse model (CGG_nih_ KI) with a CGG repeat length between 118-120CGGs by generating a CGG repeat flanked by *Sfi*I sites and then ligating this CGG repeat into the target construct with exon 1 of the mouse *Fmr1* gene in the correct orientation minimalizing changes to the mouse regions flanking the CGG repeat, the gene itself and the promoter (Entezam et al., 2007; Hoffman et al., 2012; Foote et al., 2016). This resulted in these mice having a translational TAA stop codon upstream of the CGG repeat. This TAA stop codon is present in the endogenous mouse *Fmr1* gene but not in the human *FMR1* gene. These mice, similar to the CGG_dut_ KI mouse show moderate intergenerational expansions with elevated *Fmr1* mRNA levels and decreased FMRP levels at the molecular level without any methylation of the *Fmr1* gene. Importantly, histopathological analysis revealed less ubiquitin-positive intranuclear inclusions being present in these mice compared to the CGG_dut_ KI mouse (Entezam et al., 2007; Foote et al., 2016).

## Purkinje Cell Specific Mouse Model

**FIGURE 4:**
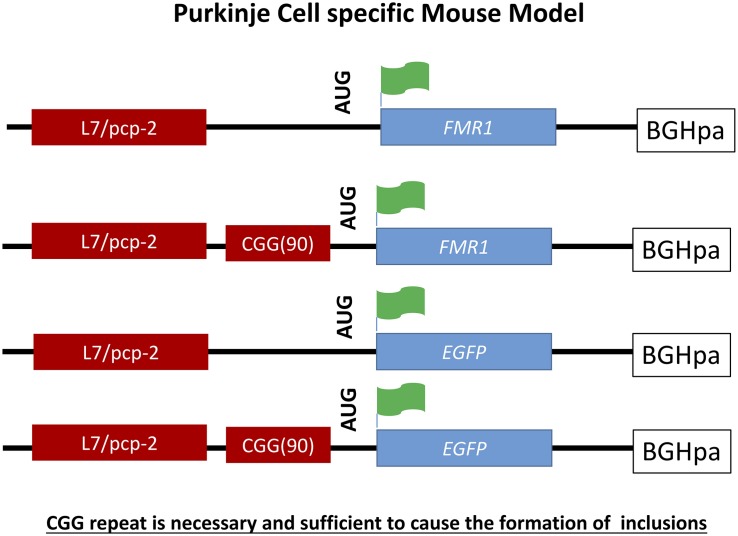
Schematic representation of L7-(CGG)90 and L7 constructs. The transgenes were driven by Purkinje cells specific, L7 promoter. A human genomic *FMR1* DNA fragment containing 90CGG repeats was inserted upstream of the *Fmr1* or EGFP coding region between the transcriptional and translational start sites. The Fmr1 containing transgenes have a FLAG epitope engineered into the 5′ region of the gene. A BGH polyadenylation site was inserted 3′ region of the transgenes.

A Purkinje cell specific mouse model was generated to provide evidence that the expanded CGG repeat is necessary to cause FXTAS pathology similar to human and distinguish these effects seen from possible alterations in the *Fmr1* gene. To do so transgenic mice were generated expressing a CGG repeat in the context of *Fmr1* (L7-CGG90-*Fmr1*) or in context of the enhanced green fluorescent protein (L7-CGG90-EGFP) specifically in Purkinje neurons using the pcp2/L7 promoter in the cerebellum (Hashem et al., 2009). With these two lines it would be possible to determine whether ectopic expression of a 90CGG expanded repeat would cause neurodegeneration in the cerebellum or not. A significant Purkinje cell loss was observed in both the L7-CGG90-*Fmr1* and L7-CGG90-EGFP mice (Figure 4). Ubiquitin-positive intranuclear inclusions being the hallmark of FXTAS could be found in Purkinje cells of both the lines with the expanded CGG repeat but not in the control mice suggesting an essential role for the expanded CGG repeat and RNA in inclusion formation. It is assumed that the proteasome degradation pathway is involved in FXTAS disease progression in humans since essential proteins involved in this pathway are found in ubiquitin-positive inclusions (Willemsen et al., 2003). Inclusions in the Purkinje cell specific mice contained as well the 20S core complex of the proteasome, heat-shock protein HSP40 and RAD23B protein, which is a known protein of the ubiquitin-mediated proteasome degradation pathway. Behavioral examination revealed that mice expressing the expanded CGG mRNA in the Purkinje cells had an impaired motor performance on the rotarod test. These neuropathological and behavioral observations provide evidence that expression of the CGG repeat mRNA is sufficient to cause Purkinje cell dysfunction and loss of neurons similar to that reported in FXTAS patients (Greco et al., 2002). Although there might be a connection between intranuclear inclusion formation and in this case Purkinje cell death, such a conclusion could only be made upon understanding the role of other essential proteins present in these inclusions.

## FMRpolyG and CGG-RNA Transgenic Mice

**FIGURE 5:**
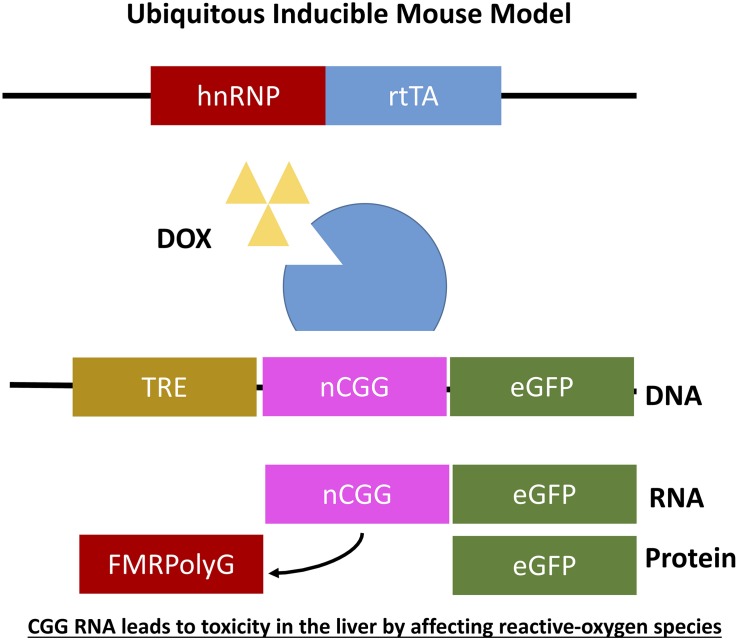
Ubiquitous expression of expanded CGG-RNA in inducible transgenic mouse model. The Tet-On system was used to generate bigenic mice expressing a expanded CGG repeat at the RNA level in all tissues. Expression of rtTA is controlled by the hnRNP promoter on a transgene. Upon DOX administration rtTA will be activated and can bind the Tet Responsive Element (TRE) on another transgene, inducing expression of the nCGG repeat at the RNA level and eGFP at the protein level.

To further elaborate on the two hypotheses regarding the pathogenic mechanism in PM and FXTAS pathology based on expression of mutant *FMR1* mRNA bearing an expanded CGG repeat a new mouse model was suggested. How *FMR1* mRNA containing expanded CGG repeats is pathogenic was still unclear. To study this, the lab of Charlet Berguerand at the Institut de Génétique et de Biologie Moléculaire et Cellulaire (IGBMC) in France created two transgenic mouse models (Figure 5). The first model contains the full human 5′ UTR of *FMR1* with expanded 99CGG repeats that express both the CGG-RNA as well as the FMRpolyG protein. The second line referred to as the mutant line also expresses 99CGG repeats, but without the non-canonical ACG start-codon and the surrounding 5′ UTR sequence region only expressing the CGG-RNA and no protein (Sellier et al., 2017). Both transgenic mouse lines have a CAG promoter inserted within the *Rosa26* locus and expression is controlled by a loxP-polyadeneylation cassette (Sellier et al., 2017). Using a ubiquitously and embryonically expressed Cre recombinase results in deletion of the loxP cassette, which leads to high expression of transgene RNA throughout the brain, heart and liver, with less expression in skeletal muscle and the kidneys. Transgene RNA expression is the same in both transgenic mouse lines. The full human *FMR1* 5′ UTR transgenic mice showed upon histological analysis FMRpolyG protein nuclear aggregates that co-localize with ubiquitin in the brain (Sellier et al., 2017). The mutant *FMR1* 5′ UTR mice did not show these aggregates. The occurrence of CGG RNA *foci* in the brain of these mice (Figure 5) is slightly lower compared to other previously reported mouse models (Sellier et al., 2010, 2017). FMRpolyG protein also accumulated in other tissue than the brain, which is similar to what our group reported regarding FMRpolyG aggregates in non-CNS tissues in FXTAS patients (Buijsen et al., 2014). Other sense proteins such as the polyalanine (FMRpolyA) and polyarginine (FMRpolyR) peptides from other reading frames were not observed in these mice. There is to some extent neuronal cell death and little loss of Purkinje neurons in the mice expressing CGG-RNA and the FMRpolyG protein but not in the mice expressing only CGG-RNA (Sellier et al., 2017).

## *FMR1* Overexpressing Mice

*FMR1* mRNA toxicity is mentioned several times before to be one of the hypothesis causing FXTAS pathology and is supported by elevated levels of *FMR1* mRNA with an expanded CGG repeat (Hagerman and Hagerman, 2002). It could be that toxicity occurs due to expression of an expanded CGG repeat or because of just elevated *FMR1* mRNA levels independent of the CGG repeat being present. A *Drosophila* model shows that repeat length is a direct cause of pathology since longer repeats show extreme retina pathology while short repeats result in little pathology (Jin et al., 2003). To confirm these findings in mice, transgenic mice were generated overexpressing *FMR1* mRNA with a normal length CGG repeat consisting of 29CGGs (Fernandez et al., 2012). Human *FMR1* cDNA with 29CGG repeats under control of a SV40/T7 promoter was injected in oocytes to generate these transgenic 29CGG mice with 20–100 fold increase in *FMR1* mRNA in tissues such as liver, cerebral cortex and cerebellum. Although these mice express extreme levels of *FMR1* mRNA, there was no significant difference in general activity or anxiety-related behaviors in open-field tests, which suggests that expression of *FMR1* mRNA alone is not enough to induce pathology but that a CGG repeat expansion is necessary for pathology to occur.

A yeast artificial chromosome (YAC) containing the full-length human *FMR1* gene was used to generate transgenic mice overexpressing *FMR1* mRNA to study the toxic effect of *FMR1* mRNA as well as to study CGG repeat instability (Peier et al., 2000; Peier and Nelson, 2002). These mice were generated by direct microinjection of purified YAC DNA containing a 92CGG repeat isolated from an adult male permutation carrier into the pro-nuclei of fertilized FVB/N mouse oocytes and then transferred into foster mice which eventually led to identifying a mouse line having a repeat containing 90CGGs (Peier and Nelson, 2002). These YAC mice show a 2–3 fold increase in expression of *FMR1* mRNA and a 10–15 fold increase in FMRP protein production (Peier et al., 2000; Spencer et al., 2008). Importantly to mention is that histological analysis presented no changes in overall brain morphology due to overexpression of *FMR1* mRNA or FMRP. When YAC mice were cross-bred with *Fmr1* KO mice lacking FMRP protein, some of the pathological features attributed to the absence of FMRP could be reversed and even overcorrected which also resulted in some abnormal behaviors. The authors attributed phenotype to overexpression of FMRP but the high levels of *FMR1* mRNA could also have contributed to these behavioral effects (Spencer et al., 2008).

## Inducible Mouse Models

**FIGURE 6:**
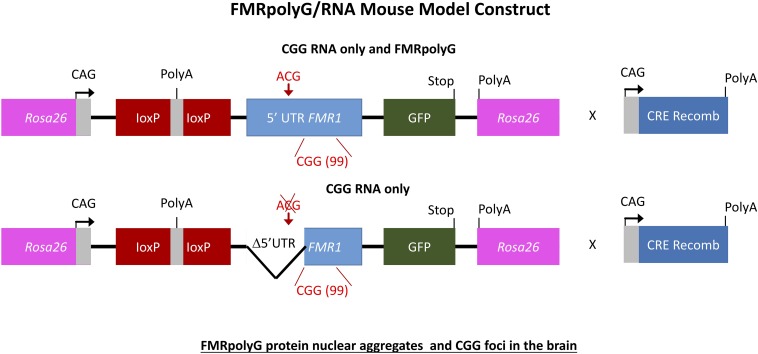
Schemes of the mouse transgene constructs. Expression of FMRpolyG is pathogentic in mice and the immunohistochemistry for FMRpolyG N-terminus of cerebellum and hippocampus areas of 6-month-old bigenic CMV-cre/full-length or mutant *FMR1* 5′-UTR transgenic mice shows occurrence of inclusions.

As mentioned before it was not yet clear whether overexpression of RNA bearing normal CGG repeat length or an expanded CGG repeat is sufficient to induce toxicity in animal models and how to translate results in mice to the PM and FXTAS pathology. To further elaborate on this question two additional doxycycline inducible transgenic mouse models were generated. Both mouse models consisted of two transgenic mouse lines differing in the promoter used. The main advantage of both models is that expression of the transgene can be switched on and off using doxycycline. The first mouse line expresses the tetracycline response element (TRE) and the other line expressing the reverse tetracycline-controlled transactivator protein (rtTA), which can be activated using doxycycline or doxycycline-derivative in drinking water or food. The TRE-element was coupled to an expanded CGG repeat in frame with eGFP while the rtTA was coupled to an hnRNP promoter. Both mouse lines were crossbred generating double transgenic offspring with ubiquitous expression of 90CGG repeat RNA (hnRNP-rtTA/TRE-90CGG-eGFP; Figure 6). Strange enough these mice died within 5 days after induction of the transgene using doxycycline. After histopathological analysis of the liver and molecular analysis two markers, cytochrome C and glutathione oxidase (GPX1), could be identified being affected in the livers with increased steatosis, mitochondrial dysfunction and subsequent apoptosis. Due to the early death of these mice no neuropathology could be observed. In summary this mouse model taught us that *in vivo* expression of expanded CGG RNA leads to severe toxicity in the liver by affecting reactive-oxygen species (ROS) signaling (Hukema et al., 2014).

Since FXTAS pathology is considered to be a neurodegenerative disease, it was clear that the next step would be to generate a brain specific doxycycline inducible mouse model expressing RNA containing an expanded CGG repeat. For this a brain specific prion protein (PrP)-rtTA driver was used to activate expression of normal length CGG RNA or expanded CGG repeat RNA fused to eGFP (Hukema et al., 2015; Castro et al., 2017). The PrP promoter drives expression in glial cells and neurons in the central nervous system (CNS). Compared to the hnRNP-rtTA mouse line, these mice could be treated for several weeks with doxycycline without any apparent liver toxicity. The RNA bearing an expanded CGG repeat was expressed the strongest in the cerebellum, hippocampus and striatum. Examining brain sections under the microscope showed ubiquitin-positive intranuclear inclusions co-localizing with FMRpolyG-positive intranuclear inclusions. Inclusion formation was followed in time showing increasing numbers and size when mice were longer treated with doxycycline. Other FXTAS-related proteins such as 20S core complex of the proteasome, HSP40 and RAD23B could also be found co-localizing with the ubiquitin-positive aggregates. Our group could show that introducing a wash-out period at an early stage was enough to halt and even reverse inclusion body formation. When repeating the wash-out step at a later point in time disease pathology could only be halted but not reversed (Hukema et al., 2015; Castro et al., 2017). Upon doxycycline induction the brain specific inducible mice had deficits in the compensatory eye movements. By turning off expression of the RNA with an expanded repeat this functional phenotype could be stopped (Hukema et al., 2015). After doxycycline induction double transgenic brain specific inducible mice performed poorly on the rotarod test indicating that motor performance is affected, more specifically motor coordination and motor learning which was also in line with the high number of intranuclear inclusions observed in lobule X of the cerebellum. When examined in the open-field test for emotional disturbance these mice were more anxious but had no deficits in emotional learning nor memory impairments correlating with intranuclear inclusions found in the amygdala and the hippocampus (Hukema et al., 2015). Behavioral phenotype and rescue were paralleled by the intranuclear inclusions formed in several brain regions such as lobule X of the cerebellum, the central amygdala and basolateral nuclei region of the amygdala, and hippocampal sub-regions such as the dentate gyrus and third cornu ammonis region of the hippocampus (Castro et al., 2017). Turning off transgene expression could halt functional phenotype and even reverse neuropathology if intervened at an early stage potentially suggesting that brain region specific therapeutic intervention might be beneficial for FXTAS patients in the future (Castro et al., 2017).

## Astrocyte-Specific Mouse Model

**FIGURE 7:**
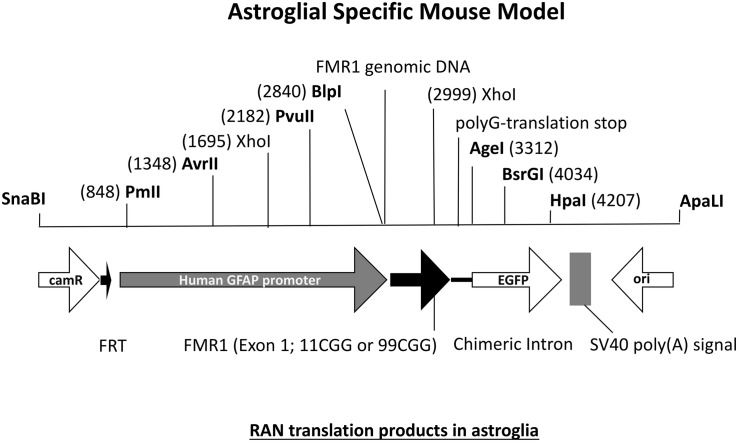
Diagram of DNA fragment used for pronuclear injection with either an 11CGG or 99CGG trinucleotide repeat expansion on exon. Astroglial-specific expression of nCGG repeat RNA yields RAN translation and formation of ubiquitin-positive and FMRpolyG-positive inclusions in astroglia and neurons.

The FXTAS field has been provided with many interesting mouse models but there are still questions left unanswered. One interesting question in the field concerns the specific role of astrocytes in FXTAS pathology. What is the role of astroglial cells in FXTAS? Is for example expression of an expanded CGG repeat enough to induce pathology? FXTAS patient brains have inclusions throughout the entire brain, in neurons but also in astroglial cells such as the Bergmann glia in the cerebellum (Willemsen et al., 2003). Although the CGG_dut_ KI mouse model of FXTAS recapitulates most aspects seen in FXTAS, these mice have relatively few numbers of astrocytes with intranuclear inclusions. Therefore an astroglial-specific mouse model was necessary. This transgenic mouse line with a C57BL/6j background was generated through pronuclear injection using a astrocyte-specific Gfa2 promoter to induce expression of an expanded 99CGG repeat fused to an eGFP marker gene only in astrocytes (Figure 7) (Wenzel et al., 2019). Immunocytochemical analysis of eGFP expression patterns show expression of 99CGG RNA was restricted to astroglia and Bergmann glia only and was not present in neurons, microglia or oligodendrocytes. Ubiquitin-positive and FMRpolyG-positive inclusions were observed in the nucleus of astroglia with inclusion bodies also being present in the cytoplasm of astrocyte processes. Although astrocytes with inclusion bodies were low in number, they were widely distributed in the brain such as in the neocortex, cerebellum and occasionally in subcortical regions in the hypothalamus and some brain stem nuclei. These mice had no sign of Purkinje neuronal dropout as well as no ubiquitin-positive inclusions in Purkinje cells could be found (Sellier et al., 2017; Wenzel et al., 2019). This study was the first of its kind to provide evidence of FXTAS related RAN-translation products being present in mouse astroglia. Surprisingly, these mice also had ubiquitin-positive intranuclear inclusions in neurons even though there was no leaky expression in neurons of the Gfa2-promoter and these cells did not express the expanded 99CGG RNA suggesting a prion-like spread of pathology, similar to what is seen in other models, from astrocytes to neurons by a cell-to-cell transfer mechanism (Westergard et al., 2016). Several behavioral tests were performed to examine neurological disease phenotypes associated with FXTAS pathology. These mice had difficulty with basic gait parameters such as stance time and range of motion. Also when placed on the ladder rung test their motor performance was clearly affected. These mice did not suffer from any emotional disturbance nor memory learning deficits. Although this model presents evidence of key hallmarks of FXTAS pathology, more work is necessary to further elaborate on the role of astroglia in FXTAS disease pathology.

## Conclusion

Different experiments have been done to study the expanded CGG repeats in the *FMR1* gene. Despite many insights, the different *in silico, in vitro*, and *in vivo* approaches have not yet resulted in a targeted therapeutic intervention for FXTAS. Indeed, while we know the exact mutation leading to disease, we still could not identify the pathogenic trigger, or combination of triggers.

The fact that a number of proteins have been consistently found with different approaches is reassuring that the synergy from different research areas give hints on their roles in pathology. For instance, studies based on RNA-pulldown (Sellier et al., 2010) and protein-microarray approaches (Cid-Samper et al., 2018) consistently show that CGG triplets, which are known to form stable hairpins *in vitro* (Krzyzosiak et al., 2012), interact physically with proteins (e.g., HNRNPs and SRSFs) that have domains annotated as preferably binding single-stranded regions (e.g., RRM domains). These results, supported by *cat*RAPID predictions (Cirillo et al., 2013), not only indicate that a number of interactions could occur in loop regions of the RNA but also suggest that the structural disorder, which is found enriched by *cat*GRANULE (Bolognesi et al., 2016) in protein partners such as TRA2A (Cid-Samper et al., 2018), might be involved in the binding, in addition to canonical domains (Castello et al., 2016). Thus, loop regions of the RNA or disordered regions of proteins could be the target for therapeutic intervention.

Candidates identified in *in silico* and *in vitro* approaches can serve as targets for intervention or as biomarkers to test the therapy can be determined by performing *in vivo* studies. For instance, the 9-hydroxy-5,11-dimethyl-2-(2-(pi-peridin-1-yl)ethyl)-6H-pyrido[4,3-b]carbazol-2-ium (also named *1a*) compound has been prioritized as a candidate for its ability to bind to loops produced by unpaired G-G nucleotides in CGG hairpins (Disney et al., 2012). The 1a compound has been used in cellular models to prevent interactions with RBPs such as TRA2A and consequent co-aggregation (Cid-Samper et al., 2018). In agreement with this finding, it has been reported that *1a* and structurally similar compounds reduce RAN translation of *FMR1* in cultured cells and patient-derived neurons (Su et al., 2014). Three small-molecule RAN inhibitors have been recently shown to interact with CGG and GGGGCC repeat RNAs by circular dichroism and native gel analysis: BIX01294, CP-31398, and propidium iodide (Green et al., 2019). These small, bioactive compounds can selectively inhibit RAN translation across multiple disease-causing repeat expansion mutations and could therefore be employed in future treatments (Green et al., 2019).

## Author Contributions

GT and TB-O wrote the *in silico* and *in vitro* section. RH and SH wrote the *in vivo* section.

## Conflict of Interest

The authors declare that the research was conducted in the absence of any commercial or financial relationships that could be construed as a potential conflict of interest. The reviewer EL declared a past co-authorship with one of the authors RH.
